# 1-Dichloro­acetyl-3,3-dimethyl-2,6-diphenyl­piperidin-4-one

**DOI:** 10.1107/S1600536808040051

**Published:** 2008-12-03

**Authors:** T. Kavitha, S. Ponnuswamy, M. Jamesh, J. Umamaheshwari, M. N. Ponnuswamy

**Affiliations:** a Centre of Advanced Study in Crystallography and Biophysics, University of Madras, Guindy Campus, Chennai 600 025, India; b Department of Chemistry, Government Arts College (Autonomous), Coimbatore 641 018, Tamil Nadu, India

## Abstract

In the title compound, C_21_H_21_Cl_2_NO_2_, the piperidine ring adopts a distorted boat conformation. The two phenyl rings are approximately perpendicular to each other, with a dihedral angle of 86.12 (7)°. Mol­ecules are linked into centrosymmetric dimers by pairs of bifurcated C—H⋯O hydrogen bonds, forming *R*_2_^2^(10) and *R*_2_^2^(14) ring motifs, and an intramolecular C—H⋯O link also occurs.

## Related literature

For general backround, see: Ponnuswamy *et al.* (2002 ▶). For details of hydrogen-bond motifs, see: Bernstein *et al.* (1995 ▶). For ring puckering and asymmetry parameters, see: Cremer & Pople (1975 ▶); Nardelli (1983 ▶). For hybridization, see: Beddoes *et al.* (1986 ▶).
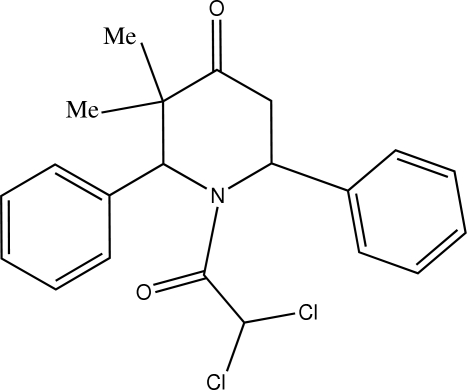


## Experimental

### 

#### Crystal data

C_21_H_21_Cl_2_NO_2_
*M* *_r_* = 390.29
Triclinic, 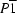

*a* = 9.1084 (2) Å
*b* = 10.8992 (3) Å
*c* = 10.9918 (3) Å
α = 63.879 (1)°
β = 85.343 (2)°
γ = 79.029 (1)°
*V* = 961.84 (4) Å^3^
*Z* = 2
Mo *K*α radiation
μ = 0.35 mm^−1^
*T* = 293 (2) K
0.30 × 0.26 × 0.20 mm

#### Data collection

Bruker Kappa APEXII area-detector diffractometer
Absorption correction: multi-scan (*SADABS*; Sheldrick, 2001 ▶) *T*_min_ = 0.902, *T*_max_ = 0.933
26977 measured reflections
7709 independent reflections
5516 reflections with *I* > 2σ(*I*)
*R*_int_ = 0.021

#### Refinement

*R*[*F* ^2^ > 2σ(*F* ^2^)] = 0.057
*wR*(*F* ^2^) = 0.180
*S* = 1.03
7709 reflections
235 parameters
H-atom parameters constrained
Δρ_max_ = 0.80 e Å^−3^
Δρ_min_ = −0.73 e Å^−3^

### 

Data collection: *APEX2* (Bruker, 2004 ▶); cell refinement: *APEX2*; data reduction: *SAINT* (Bruker, 2004 ▶); program(s) used to solve structure: *SHELXS97* (Sheldrick, 2008 ▶); program(s) used to refine structure: *SHELXL97* (Sheldrick, 2008 ▶); molecular graphics: *PLATON* (Spek, 2003 ▶); software used to prepare material for publication: *SHELXL97*.

## Supplementary Material

Crystal structure: contains datablocks I, global. DOI: 10.1107/S1600536808040051/ci2724sup1.cif

Structure factors: contains datablocks I. DOI: 10.1107/S1600536808040051/ci2724Isup2.hkl

Additional supplementary materials:  crystallographic information; 3D view; checkCIF report

## Figures and Tables

**Table 1:** Hydrogen-bond geometry (Å, °)

*D*—H⋯*A*	*D*—H	H⋯*A*	*D*⋯*A*	*D*—H⋯*A*
C14—H14⋯O2	0.93	2.57	3.250 (2)	130
C2—H2⋯O2^i^	0.98	2.50	3.4264 (18)	158
C16—H16*A*⋯O2^i^	0.96	2.54	3.413 (2)	151

Symmetry code: (i) 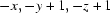.

**Fig. 1.**
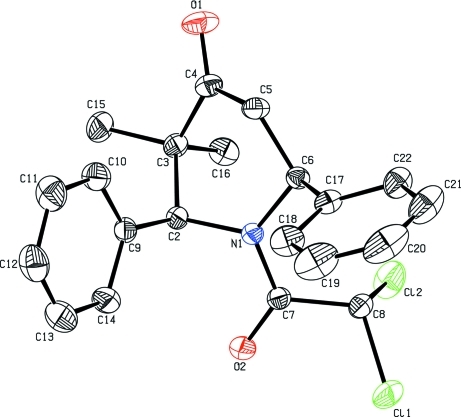
The molecular structure of the title compound. Displacement ellipsoids are drawn at the 30% probability level. H atoms have been omitted for clarity.

**Fig. 2.**
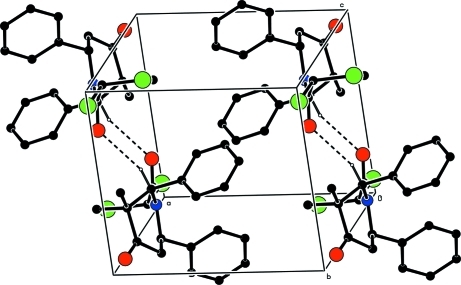
The crystal packing of the title compound, showing hydrogen-bonded (dashed lines) dimers. Atom H16A and H atoms not involved in hydrogen bonding have been omitted.

## supplementary crystallographic information

### Comment

The design and synthesis of conformationally anchored molecules are important due its potency and selectivity for designing drugs. The piperidin-4-ones are one such class of compounds to be investigated to understand the stereodynamics and other structural features (Ponnuswamy *et al.*, 2002).

The sum of bond angles around atom N1 (359.6°) indicates *sp*^2^ hybridization (Beddoes *et al.*, 1986). The N1—C7 [1.3564 (16) Å] and C7—O2 [1.2149 (17) Å] distances indicate electron delocalization. The piperidine ring adopts a distorted boat conformation, with puckering parameters (Cremer & Pople, 1975) q2 = 0.638 (1) Å, q3 = -0.067 (2) Å and φ_2_ = 253.4 (1)°, and the asymmetry parameters ΔC_s_(C2)= 14.4 (1)° (Nardelli, 1983). The best plane through the piperidine ring, N1/C3/C4/C6, forms dihedral angles of 89.31 (6)° and 63.47 (7)°, respectively, with the C9—C4 and C17—C22 phenyl rings. The two phenyl rings are approximately perpendicular to each other, with a dihedral angle of 86.12 (7)°.

The crystal structure is stabilized by intermolecular C—H···O hydrogen bonds. Each atoms C2 and C16 at (*x*, *y*, *z*) donate one proton to bifurcated acceptor O2 at (-*x*, 1 - *y*, 1 - *z*), forming a centrosymmetric dimer (Fig. 2) with *R*_2_^2^(10) and *R*_2_^2^(14) ring motifs (Bernstein *et al.*, 1995).

### Experimental

A mixture of 3,3-dimethyl-*cis*-2,6-diphenylpiperidin-4-one (1.4 g, 5 mmol), dichloroacetylchloride (1 ml, 10 mmol) and triethylamine (2 ml, 14.4 mmol) in anhydrous benzene (20 ml) was stirred at room temperature for 7 h. The benzene solution was dried over anhydrous Na_2_SO_4_ and concentrated. The pasty mass obtained was purified by crystallization from benzene-petroleum ether (333–353 K) in the ratio of 95:5.

### Refinement

H atoms were positioned geometrically (C—H = 0.93–0.98 Å) and allowed to ride on their parent atoms, with *U*_iso_(H) = 1.5*U*_eq_(C) for methyl H and 1.2*U*_eq_(C) for other H atoms.

### Crystal data

**Table:**

C_21_H_21_Cl_2_NO_2_	*Z* = 2
*M**_r_* = 390.29	*F*(000) = 408
Triclinic, *P*1	*D*_x_ = 1.348 Mg m^−^^3^
Hall symbol: -P 1	Mo *K*α radiation, λ = 0.71073 Å
*a* = 9.1084 (2) Å	Cell parameters from 7709 reflections
*b* = 10.8992 (3) Å	θ = 2.1–34.0°
*c* = 10.9918 (3) Å	µ = 0.35 mm^−^^1^
α = 63.879 (1)°	*T* = 293 K
β = 85.343 (2)°	Block, colourless
γ = 79.029 (1)°	0.30 × 0.26 × 0.20 mm
*V* = 961.84 (4) Å^3^	

### Data collection

**Table:**

Bruker Kappa APEXII area-detector diffractometer	7709 independent reflections
Radiation source: fine-focus sealed tube	5516 reflections with *I* > 2σ(*I*)
graphite	*R*_int_ = 0.021
ω and φ scans	θ_max_ = 34.0°, θ_min_ = 2.1°
Absorption correction: multi-scan (*SADABS*; Sheldrick, 2001)	*h* = −14→14
*T*_min_ = 0.902, *T*_max_ = 0.933	*k* = −16→17
26977 measured reflections	*l* = −17→15

### Refinement

**Table:**

Refinement on *F*^2^	Primary atom site location: structure-invariant direct methods
Least-squares matrix: full	Secondary atom site location: difference Fourier map
*R*[*F*^2^ > 2σ(*F*^2^)] = 0.057	Hydrogen site location: inferred from neighbouring sites
*wR*(*F*^2^) = 0.180	H-atom parameters constrained
*S* = 1.03	*w* = 1/[σ^2^(*F*_o_^2^) + (0.0854*P*)^2^ + 0.3205*P*] where *P* = (*F*_o_^2^ + 2*F*_c_^2^)/3
7709 reflections	(Δ/σ)_max_ = 0.001
235 parameters	Δρ_max_ = 0.80 e Å^−^^3^
0 restraints	Δρ_min_ = −0.73 e Å^−^^3^

### Special details

**Table:**

Geometry. All e.s.d.'s (except the e.s.d. in the dihedral angle between two l.s. planes) are estimated using the full covariance matrix. The cell e.s.d.'s are taken into account individually in the estimation of e.s.d.'s in distances, angles and torsion angles; correlations between e.s.d.'s in cell parameters are only used when they are defined by crystal symmetry. An approximate (isotropic) treatment of cell e.s.d.'s is used for estimating e.s.d.'s involving l.s. planes.
Refinement. Refinement of *F*^2^ against ALL reflections. The weighted *R*-factor *wR* and goodness of fit *S* are based on *F*^2^, conventional *R*-factors *R* are based on *F*, with *F* set to zero for negative *F*^2^. The threshold expression of *F*^2^ > σ(*F*^2^) is used only for calculating *R*-factors(gt) *etc*. and is not relevant to the choice of reflections for refinement. *R*-factors based on *F*^2^ are statistically about twice as large as those based on *F*, and *R*- factors based on ALL data will be even larger.

### Fractional atomic coordinates and isotropic or equivalent isotropic displacement parameters (Å^2^)

**Table:**

	*x*	*y*	*z*	*U*_iso_*/*U*_eq_	
C2	0.16315 (15)	0.26040 (13)	0.64530 (13)	0.0320 (2)	
H2	0.1059	0.3371	0.5691	0.038*	
C3	0.07185 (16)	0.14160 (15)	0.69561 (15)	0.0363 (3)	
C4	0.12824 (17)	0.02567 (15)	0.83179 (16)	0.0394 (3)	
C5	0.22508 (18)	0.06135 (14)	0.91179 (15)	0.0392 (3)	
H5A	0.3283	0.0437	0.8854	0.047*	
H5B	0.2169	−0.0007	1.0070	0.047*	
C6	0.18871 (15)	0.21096 (13)	0.89554 (13)	0.0315 (2)	
H6	0.0976	0.2204	0.9469	0.038*	
C7	0.11626 (15)	0.44855 (13)	0.71233 (13)	0.0330 (2)	
C8	0.06652 (16)	0.49415 (14)	0.82515 (15)	0.0369 (3)	
H8	0.1194	0.4282	0.9094	0.044*	
C9	0.32045 (16)	0.23856 (15)	0.59084 (14)	0.0362 (3)	
C10	0.4137 (2)	0.11132 (19)	0.62714 (19)	0.0521 (4)	
H10	0.3796	0.0306	0.6872	0.062*	
C11	0.5572 (2)	0.1029 (3)	0.5750 (2)	0.0641 (5)	
H11	0.6186	0.0167	0.6012	0.077*	
C12	0.6095 (2)	0.2197 (3)	0.4854 (2)	0.0646 (5)	
H12	0.7062	0.2136	0.4516	0.078*	
C13	0.5178 (3)	0.3460 (3)	0.4460 (2)	0.0683 (6)	
H13	0.5521	0.4258	0.3842	0.082*	
C14	0.3742 (2)	0.35549 (19)	0.49753 (19)	0.0522 (4)	
H14	0.3129	0.4419	0.4690	0.063*	
C15	0.0667 (2)	0.0880 (2)	0.5892 (2)	0.0554 (4)	
H15A	0.0305	0.1640	0.5050	0.083*	
H15B	0.0010	0.0210	0.6192	0.083*	
H15C	0.1654	0.0452	0.5765	0.083*	
C16	−0.09026 (17)	0.19822 (18)	0.72274 (18)	0.0458 (3)	
H16A	−0.1334	0.2725	0.6403	0.069*	
H16B	−0.0895	0.2320	0.7899	0.069*	
H16C	−0.1485	0.1252	0.7549	0.069*	
C17	0.31594 (16)	0.23865 (14)	0.95568 (15)	0.0377 (3)	
C18	0.4438 (2)	0.2760 (2)	0.8816 (2)	0.0564 (4)	
H18	0.4525	0.2854	0.7932	0.068*	
C19	0.5588 (3)	0.2993 (3)	0.9398 (3)	0.0812 (8)	
H19	0.6448	0.3239	0.8903	0.097*	
C20	0.5464 (3)	0.2865 (3)	1.0703 (3)	0.0855 (9)	
H20	0.6232	0.3041	1.1080	0.103*	
C21	0.4221 (3)	0.2479 (3)	1.1444 (3)	0.0749 (7)	
H21	0.4148	0.2384	1.2329	0.090*	
C22	0.3049 (2)	0.22256 (19)	1.08834 (19)	0.0523 (4)	
H22	0.2206	0.1952	1.1396	0.063*	
Cl1	0.09918 (6)	0.66177 (5)	0.78274 (6)	0.06072 (15)	
Cl2	−0.12724 (6)	0.49086 (7)	0.84645 (7)	0.07096 (18)	
N1	0.16105 (12)	0.31050 (11)	0.75154 (11)	0.0301 (2)	
O1	0.09414 (17)	−0.08818 (13)	0.87624 (15)	0.0596 (4)	
O2	0.10590 (15)	0.53523 (11)	0.59509 (11)	0.0479 (3)	

### Atomic displacement parameters (Å^2^)

**Table:**

	*U*^11^	*U*^22^	*U*^33^	*U*^12^	*U*^13^	*U*^23^
C2	0.0360 (6)	0.0327 (5)	0.0303 (5)	−0.0091 (5)	0.0005 (4)	−0.0151 (5)
C3	0.0389 (7)	0.0372 (6)	0.0392 (6)	−0.0133 (5)	0.0001 (5)	−0.0198 (5)
C4	0.0407 (7)	0.0324 (6)	0.0470 (7)	−0.0117 (5)	0.0009 (6)	−0.0170 (5)
C5	0.0463 (7)	0.0291 (6)	0.0386 (7)	−0.0096 (5)	−0.0051 (6)	−0.0094 (5)
C6	0.0327 (6)	0.0314 (5)	0.0299 (5)	−0.0085 (4)	−0.0015 (4)	−0.0113 (4)
C7	0.0356 (6)	0.0300 (5)	0.0337 (6)	−0.0068 (5)	−0.0007 (5)	−0.0134 (5)
C8	0.0397 (7)	0.0334 (6)	0.0399 (7)	−0.0043 (5)	−0.0015 (5)	−0.0186 (5)
C9	0.0390 (7)	0.0403 (6)	0.0343 (6)	−0.0114 (5)	0.0046 (5)	−0.0196 (5)
C10	0.0480 (9)	0.0468 (8)	0.0557 (9)	−0.0050 (7)	0.0120 (7)	−0.0203 (7)
C11	0.0477 (10)	0.0720 (13)	0.0679 (12)	0.0016 (9)	0.0097 (9)	−0.0323 (10)
C12	0.0434 (9)	0.0902 (16)	0.0660 (12)	−0.0188 (10)	0.0178 (8)	−0.0393 (11)
C13	0.0647 (12)	0.0741 (13)	0.0692 (13)	−0.0349 (11)	0.0304 (10)	−0.0298 (11)
C14	0.0571 (10)	0.0460 (8)	0.0521 (9)	−0.0181 (7)	0.0171 (8)	−0.0193 (7)
C15	0.0692 (12)	0.0611 (10)	0.0551 (10)	−0.0264 (9)	0.0026 (8)	−0.0368 (9)
C16	0.0356 (7)	0.0511 (8)	0.0496 (8)	−0.0138 (6)	−0.0013 (6)	−0.0183 (7)
C17	0.0371 (6)	0.0323 (6)	0.0439 (7)	−0.0035 (5)	−0.0108 (5)	−0.0158 (5)
C18	0.0418 (8)	0.0620 (11)	0.0710 (12)	−0.0179 (7)	−0.0038 (8)	−0.0296 (9)
C19	0.0471 (11)	0.0829 (16)	0.128 (2)	−0.0202 (10)	−0.0174 (12)	−0.0520 (16)
C20	0.0652 (14)	0.0734 (14)	0.136 (2)	0.0039 (11)	−0.0552 (16)	−0.0588 (16)
C21	0.0874 (16)	0.0676 (12)	0.0810 (14)	0.0157 (11)	−0.0504 (13)	−0.0454 (11)
C22	0.0588 (10)	0.0522 (9)	0.0486 (9)	0.0007 (7)	−0.0185 (7)	−0.0254 (7)
Cl1	0.0769 (3)	0.0499 (2)	0.0729 (3)	−0.0258 (2)	0.0075 (2)	−0.0378 (2)
Cl2	0.0479 (3)	0.0867 (4)	0.1055 (5)	−0.0242 (2)	0.0255 (3)	−0.0656 (4)
N1	0.0339 (5)	0.0282 (4)	0.0290 (5)	−0.0072 (4)	−0.0003 (4)	−0.0124 (4)
O1	0.0717 (9)	0.0355 (5)	0.0709 (8)	−0.0231 (6)	−0.0097 (7)	−0.0151 (6)
O2	0.0706 (8)	0.0324 (5)	0.0350 (5)	−0.0071 (5)	−0.0009 (5)	−0.0102 (4)

### Geometric parameters (Å, °)

**Table:**

C2—N1	1.4886 (17)	C11—C12	1.367 (3)
C2—C9	1.525 (2)	C11—H11	0.93
C2—C3	1.5424 (19)	C12—C13	1.369 (3)
C2—H2	0.98	C12—H12	0.93
C3—C4	1.521 (2)	C13—C14	1.387 (3)
C3—C15	1.528 (2)	C13—H13	0.93
C3—C16	1.547 (2)	C14—H14	0.93
C4—O1	1.2096 (18)	C15—H15A	0.96
C4—C5	1.503 (2)	C15—H15B	0.96
C5—C6	1.5324 (19)	C15—H15C	0.96
C5—H5A	0.97	C16—H16A	0.96
C5—H5B	0.97	C16—H16B	0.96
C6—N1	1.4814 (16)	C16—H16C	0.96
C6—C17	1.5179 (18)	C17—C18	1.385 (3)
C6—H6	0.98	C17—C22	1.387 (2)
C7—O2	1.2149 (17)	C18—C19	1.387 (3)
C7—N1	1.3564 (16)	C18—H18	0.93
C7—C8	1.535 (2)	C19—C20	1.375 (4)
C8—Cl1	1.7544 (15)	C19—H19	0.93
C8—Cl2	1.7664 (16)	C20—C21	1.361 (4)
C8—H8	0.98	C20—H20	0.93
C9—C14	1.386 (2)	C21—C22	1.401 (3)
C9—C10	1.387 (2)	C21—H21	0.93
C10—C11	1.388 (3)	C22—H22	0.93
C10—H10	0.93		
			
N1—C2—C9	111.62 (11)	C10—C11—H11	119.6
N1—C2—C3	108.79 (10)	C11—C12—C13	119.27 (18)
C9—C2—C3	119.05 (12)	C11—C12—H12	120.4
N1—C2—H2	105.4	C13—C12—H12	120.4
C9—C2—H2	105.4	C12—C13—C14	120.42 (19)
C3—C2—H2	105.4	C12—C13—H13	119.8
C4—C3—C15	112.03 (13)	C14—C13—H13	119.8
C4—C3—C2	111.80 (11)	C9—C14—C13	121.16 (18)
C15—C3—C2	111.26 (13)	C9—C14—H14	119.4
C4—C3—C16	104.84 (12)	C13—C14—H14	119.4
C15—C3—C16	108.17 (14)	C3—C15—H15A	109.5
C2—C3—C16	108.41 (12)	C3—C15—H15B	109.5
O1—C4—C5	121.20 (14)	H15A—C15—H15B	109.5
O1—C4—C3	122.41 (14)	C3—C15—H15C	109.5
C5—C4—C3	116.35 (11)	H15A—C15—H15C	109.5
C4—C5—C6	115.68 (12)	H15B—C15—H15C	109.5
C4—C5—H5A	108.4	C3—C16—H16A	109.5
C6—C5—H5A	108.4	C3—C16—H16B	109.5
C4—C5—H5B	108.4	H16A—C16—H16B	109.5
C6—C5—H5B	108.4	C3—C16—H16C	109.5
H5A—C5—H5B	107.4	H16A—C16—H16C	109.5
N1—C6—C17	112.02 (11)	H16B—C16—H16C	109.5
N1—C6—C5	110.99 (11)	C18—C17—C22	119.73 (16)
C17—C6—C5	108.73 (11)	C18—C17—C6	121.13 (14)
N1—C6—H6	108.3	C22—C17—C6	119.12 (15)
C17—C6—H6	108.3	C17—C18—C19	119.8 (2)
C5—C6—H6	108.3	C17—C18—H18	120.1
O2—C7—N1	124.28 (13)	C19—C18—H18	120.1
O2—C7—C8	119.02 (12)	C20—C19—C18	120.5 (3)
N1—C7—C8	116.59 (11)	C20—C19—H19	119.8
C7—C8—Cl1	111.99 (10)	C18—C19—H19	119.8
C7—C8—Cl2	106.14 (10)	C21—C20—C19	120.06 (19)
Cl1—C8—Cl2	109.75 (8)	C21—C20—H20	120.0
C7—C8—H8	109.6	C19—C20—H20	120.0
Cl1—C8—H8	109.6	C20—C21—C22	120.6 (2)
Cl2—C8—H8	109.6	C20—C21—H21	119.7
C14—C9—C10	117.55 (15)	C22—C21—H21	119.7
C14—C9—C2	117.21 (14)	C17—C22—C21	119.4 (2)
C10—C9—C2	125.24 (13)	C17—C22—H22	120.3
C9—C10—C11	120.81 (18)	C21—C22—H22	120.3
C9—C10—H10	119.6	C7—N1—C6	122.10 (11)
C11—C10—H10	119.6	C7—N1—C2	116.79 (10)
C12—C11—C10	120.7 (2)	C6—N1—C2	120.69 (10)
C12—C11—H11	119.6		
			
N1—C2—C3—C4	−55.28 (15)	C11—C12—C13—C14	0.8 (4)
C9—C2—C3—C4	74.08 (15)	C10—C9—C14—C13	−2.2 (3)
N1—C2—C3—C15	178.63 (13)	C2—C9—C14—C13	177.88 (18)
C9—C2—C3—C15	−52.01 (18)	C12—C13—C14—C9	0.7 (3)
N1—C2—C3—C16	59.80 (14)	N1—C6—C17—C18	−39.86 (19)
C9—C2—C3—C16	−170.84 (12)	C5—C6—C17—C18	83.18 (17)
C15—C3—C4—O1	−38.8 (2)	N1—C6—C17—C22	141.83 (14)
C2—C3—C4—O1	−164.51 (16)	C5—C6—C17—C22	−95.13 (16)
C16—C3—C4—O1	78.24 (19)	C22—C17—C18—C19	−1.1 (3)
C15—C3—C4—C5	143.33 (15)	C6—C17—C18—C19	−179.38 (18)
C2—C3—C4—C5	17.66 (18)	C17—C18—C19—C20	−0.4 (4)
C16—C3—C4—C5	−99.59 (15)	C18—C19—C20—C21	1.3 (4)
O1—C4—C5—C6	−144.98 (16)	C19—C20—C21—C22	−0.7 (4)
C3—C4—C5—C6	32.88 (19)	C18—C17—C22—C21	1.6 (3)
C4—C5—C6—N1	−43.61 (17)	C6—C17—C22—C21	179.98 (15)
C4—C5—C6—C17	−167.26 (13)	C20—C21—C22—C17	−0.7 (3)
O2—C7—C8—Cl1	−33.06 (17)	O2—C7—N1—C6	172.74 (13)
N1—C7—C8—Cl1	150.79 (10)	C8—C7—N1—C6	−11.34 (18)
O2—C7—C8—Cl2	86.69 (15)	O2—C7—N1—C2	−14.7 (2)
N1—C7—C8—Cl2	−89.46 (13)	C8—C7—N1—C2	161.25 (11)
N1—C2—C9—C14	−78.53 (17)	C17—C6—N1—C7	−63.04 (16)
C3—C2—C9—C14	153.41 (15)	C5—C6—N1—C7	175.21 (12)
N1—C2—C9—C10	101.51 (17)	C17—C6—N1—C2	124.65 (13)
C3—C2—C9—C10	−26.6 (2)	C5—C6—N1—C2	2.90 (16)
C14—C9—C10—C11	2.2 (3)	C9—C2—N1—C7	99.87 (13)
C2—C9—C10—C11	−177.88 (17)	C3—C2—N1—C7	−126.77 (12)
C9—C10—C11—C12	−0.7 (3)	C9—C2—N1—C6	−87.43 (14)
C10—C11—C12—C13	−0.8 (4)	C3—C2—N1—C6	45.93 (15)

### Hydrogen-bond geometry (Å, °)

**Table:**

*D*—H···*A*	*D*—H	H···*A*	*D*···*A*	*D*—H···*A*
C14—H14···O2	0.93	2.57	3.250 (2)	130
C2—H2···O2^i^	0.98	2.50	3.4264 (18)	158
C16—H16A···O2^i^	0.96	2.54	3.413 (2)	151

Symmetry codes: (i) −*x*, −*y*+1, −*z*+1.
